# Nutritional and Oral Hygiene Knowledge versus Reported Behavior of Children and Adolescents—A Cross-Sectional Interview-Based Study

**DOI:** 10.3390/ijerph191610055

**Published:** 2022-08-15

**Authors:** Madline P. Gund, Carola Unshelm, Matthias Hannig, Stefan Rupf

**Affiliations:** 1 Clinic of Operative Dentistry, Periodontology and Preventive Dentistry, Saarland University, D-66421 Homburg, Germany; 2 Synoptic Dentistry, Saarland University, D-66421 Homburg, Germany

**Keywords:** dental caries, obesity, overweight, nutritional knowledge, nutritional behavior, healthy nutrition, oral hygiene, oral hygiene knowledge, oral hygiene behavior

## Abstract

Despite the fact that healthy, sugar-reduced nutrition has been propagated by the media and in schools for years, dental caries in children remains a major health problem worldwide, caused primarily by an unhealthy diet. The objective of this study is to compare statements on nutrition and hygienic knowledge with those on children’s actual dietary and hygienic behavior. A random sample of 554 children and adolescents aged 3–19 years was enrolled. Designed as a cross-sectional interview-based community survey, this study was conducted consecutively during three one-day public science-promoting events at the Saarland University’s Medical Faculty. Participants’ oral hygiene and nutritional knowledge was profound; however, the reported translation into practice showed deficiencies. Boys and younger children (3–10 years) often showed better oral hygiene knowledge than girls and older children (over 11 years) but had problems implementing it into their daily life practice. In contrast, girls and older children often showed less oral hygiene knowledge but reported more favorable behavior. Finally, children up to the mixed dentition phase preferred drinking sweets more often than older children, posing a risk to the developing permanent dentition. Intensifying well-known controlled motivation training approaches to encourage children and adolescents is recommended to put their knowledge into practice.

## 1. Introduction

Unhealthy nutritional behavior (the consumption of fat and sweet food, sweet drinks, and soft drinks) has many consequences, leading to major problems such as obesity, overweight and dental caries. 

Dental caries in children is one of the most frequent diseases worldwide [1]. The caries process is induced by the demineralization of the enamel. The main factor is organic acids forming microorganisms in the dysbiotic plaque biofilm, metabolizing fermentable carbohydrates from foods [2]. Early childhood caries is defined as caries in children of pre-school age in the first dentition [3], often proceeding aggressively. The risk for early caries is increased if the child falls asleep with a filled bottle in the mouth due to reduced flow during sleep [4]. Untreated caries may affect smile, speech, mastication function, and quality of life. Furthermore, it may lead to pain, abscesses, swelling and psychological disturbances. Treatment is expensive and prevention is simple [5]. Commonly known oral hygiene measures are brushing, the daily use of fluoride toothpaste, interdental floss/brushes, and additional fluoridation with a mouth rinse or fluoride gel [6,7]. Like caries, obesity is a consequence of an unhealthy diet. Children with early childhood caries often suffer from obesity [8]. Obesity and overweight frequently affect children worldwide. In 2016, 18% of 5–9-year-old children and adolescents suffered from obesity or overweight [9]. A review, published in 2021, confirmed sports, diet, and a change in behavior to be effective against obesity and overweight [10]. Programs fighting against poor nutritional behavior are in progress. Recently, the WHO Regional Committee for Europe initiated a European Program of Work (EPW), starting in 2020 and ending in 2025, aiming to foster better health in Europe. Fighting obesity and promoting sports and healthy nutritional behavior are part of the EPW [11]. In this respect, synergies might be used in the effort to improve caries prevention and the fight against obesity.

Knowing poor nutritional behavior is still a problem and healthy nutritional knowledge is common nowadays, this cross-sectional interview-based analysis aims to find out whether there is a discrepancy between nutritional knowledge and behavior. The question is investigated by examining nutritional knowledge and the reported behavior of children and adolescents, assuming knowledge and behavior do not differ.

## 2. Materials and Methods

### 2.1. Study Design, Participants and Setting

This cross-sectional questionnaire-based, two-part interview-style study was conducted at a German University Medical Centre from 2016 to 2018. The questionnaire aimed to record the participants’ nutritional and oral hygiene knowledge and their self-reported behavior. The age and gender of the participants were recorded. The participants were interviewed only once. Repeated participation was excluded by questioning. The participant’s identifiable data were not collected. Children, adolescents, and young adults (CAYA) aged 3–19 years that attended the “Long Night of Sciences” event at Saarland University´s Medical Faculty in 2016, 2017 and 2018 were consecutively enrolled as participants. The sample size could not be derived, as in each case it was impossible to predict how many children would participate per year.

Two age groups were formed: children with primary and mixed dentition (3–10 years) and permanent dentition (over 11 years), commonly referred to as younger children (3–10 years) and older children (over 11 years). The refusal of participation or missing parental consent were the only reasons for exclusion. Students helped to interview the children.

### 2.2. Nutritional Knowledge and Self-Reported Nutritional Behaviorethics

The answers were predefined in both parts of the questionnaire. The first part addressed nutritional knowledge. The second part recorded the self-reported behavior. Both questionnaires recorded tooth brushing frequency and oral hygiene behavior after eating candy and drinking sweet liquids. The questionnaire was validated in 2016 with 93/554 CAYAs completing it (see Questionnaire S1 in Supplementary Material). In 2016, the knowledge and behavior sections were included in one questionnaire with the same participants answering both parts. In 2017 and 2018 the knowledge and behavior sections were separated with the aim to interview different CAYAs in the two different sections to avoid too-similar knowledge answers and self-reported behavior, as detected in 2016. Furthermore, after the validation and review in 2016, not all questions were asked again in 2017 and 2018. The questions were removed if the recorded answers were not clear or productive. Additionally, after reviewing the results of 2016, some nutritional knowledge questions were added in the following years which were missing in 2016, completing the overall impression. The final questionnaire version (see Questionnaire S2 in Supplementary Material) was validated in 2017, with 78/554 (part knowledge) and 75/554 CAYAs (part behavior) confirming the results previously recorded. The CAYAs independent answers were monitored, and parental involvement was only approved for children aged 3–5 years to help them. 

### 2.3. Data Analysis

The data analysis utilized IBM SPSS 28 (IBM Deutschland GmbH, Ehningen, Germany). The responses to the questions were evaluated generally and pooled for ages (3–10, over 11) and gender (male/female). 

The Pearson correlation was checked using the R software. Using the cor.test function, a *p*-value for the Pearson correlation could be assessed. Additionally, non-parametric permutation tests were carried out as standard controls to estimate whether the correlation was random or not. This test has the strength of not making assumptions on the distribution of data, and thus is well suited to cross-check the results of the parametric Pearson correlation. 

In more detail, 1000 random permutations of the variables were performed and computed with the fraction of permutations reaching a higher correlation value (f). The *p*-value was then computed as *p* = f/1000. If less than 50 random permutations reached a higher correlation, the result was considered significant at an alpha level of 0.05. 

## 3. Result

Nearly all participants self-reported the daily consumption of sweets (98.7%) with 68.9% in the knowledge section stating this was correct. Girls and boys were making nearly similar statements regarding knowledge and behavior, though boys were slightly better informed (daily consumption knowledge: boys, 63.1%; girls, 70.5%). Children in the first and mixed dentition phase (3–10 years) preferred eating sweets several times a day (99%), posing a potential risk for a caries-prone first dentition. Older children (over 11 years) self-reported eating nearly the same amount (100%). Younger children showed better knowledge than older children (every-day consumption: older children, 76.2%; younger children, 68.7%). 

Fruits were considered most frequently (68.6%) as a suitable, healthy snack. This was confirmed in all age and gender groups. However, participants who considered sweets as a suitable snack increased over the years from 4.3% in 2016 to 10.9% in 2018. On the other side, only 39.8% of participants stated the consumption of fruits as a snack with a clear discrepancy between the behavior of boys (23.7%) and girls (56.4%) and between the age groups (3–10 years, 39.6%; from 11 years, 20%). In the end, 25.8% self-reported eating sweets as a snack with only 8.2% stating that this was correct. Boys ate more sweets than girls (31.8%; 20.5%). Older children self-reported eating more sweets than younger children (30%; 24.5%) and less fruits (20%; 39.6%), despite being better informed (knowledge section: sweets as snack: 1.2%, older children; 8.2%, younger children).

Two trends emerged in the frequency of snack consumption: one group consuming always/usually (44.9%, knowledge: 48.1%) and another group sometimes/seldom (52.4%, knowledge: 49.4%). No major differences between age and gender could be detected in these two groups. Girls tended to consume snacks more frequently than boys, who were more cautious (seldom-behavior: 54.5%; always: 41.3%) and better informed (seldom-knowledge: boys 54.8%; girls 45.7%; always/usually-knowledge: boys 43.8%; girls 52.8%). 

Sugar-sweetened beverages (SBBs) were by far the most consumed (67.1%; water/(unsweetened) tea, 29.8%). In contrast, the majority stated that water and tea should be consumed (74.5%) with the over-11-year-olds affirming water and tea consumption the most frequently (87.5%). Boys self-reported drinking more SBBs (71.1%) than girls (65.4%) and these groups in turn self-reported drinking more water and tea (32.1%), though knowledge was nearly the same in both groups. Older children preferred healthier drinks (over 11 years: SBBs, 60.4%; water/tea, 37.7%) than younger ones (SBBs, 71.1%; water/tea, 25.5%), posing a potential risk of caries-prone first dentition. Furthermore, older children were better informed than younger children (SBB consumption: over 11 years-knowledge, 12.5%; 3–10 years-knowledge, 23.9%). Generally, a clear discrepancy between knowledge and behavior was recorded and SBB consumption was far too high.

The majority claimed to drink sweet drinks (65.5%) less than every day with 74.5% indicating in the knowledge section that this was correct. Nevertheless, 34.2% of the participants still self-reported drinking sweet drinks every day with 24.3% affirming this behavior in the knowledge section. Girls and children over 11 years tended to drink sweet drinks less frequently than every day (girls, 72.3%; older children, 69.8%). A clear discrepancy between boys and girls was found in their behavior, though no great differences could be detected in the knowledge section (every day: boys-behavior, 43%; girls-behavior, 27%; boys-knowledge, 24.7%; girls-knowledge, 24.4%). In the knowledge section, older children most frequently indicated the daily consumption of sweet drinks as correct (34.4%; the least frequently indicated was in 3-10-year-olds, 21.1%) and least frequently identified non-daily consumption (65.7%; the most frequently indicated was in 3-10-year-olds, 78.1%). The knowledge and behavior of girls and older children matched most consistently.

While a large majority favored brushing teeth after eating sweets (70.4%) and a minority doing nothing (5.5%), no special oral hygiene measures were conducted (58.6%) in daily practice in the majority. Younger children and boys seemed to have better knowledge about dental hygiene behavior after sweet consumption (brushing teeth afterwards: younger children and boys, 73.9%) than older children (59.5%) and girls (68.7%). Boys also favored chewing gum more often (13.5%; girls, 6.6%) and negated doing nothing (boys, 3.6%; girls, 6.0%). Nevertheless, in daily practice both girls and older children behaved more healthily (brushing teeth: girls, 17.6%; boys, 14.1%; older children, 17%; younger children, 15.7%). Furthermore, boys and younger children more often self-reported doing nothing after sweets consumption (boys, 65.3%; girls, 56.6%; older children, 61.3%; younger children, 54.7%). Generally, knowledge and daily practice behavior were not congruent in our study.

**Figure 1:**
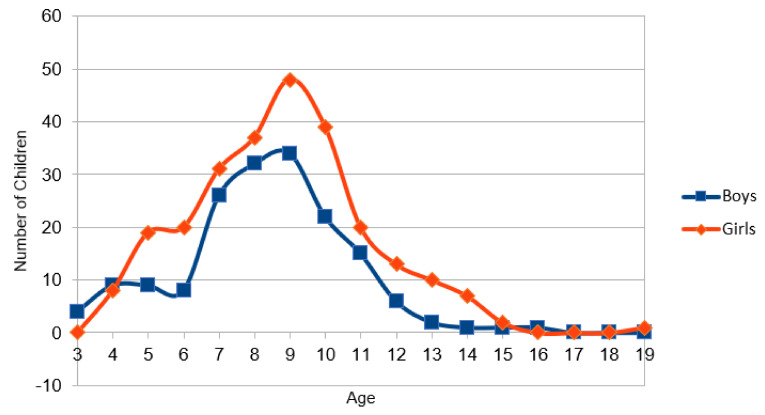
Age and gender distribution across all years (2016, 2017, 2018).

A slim majority favored brushing teeth three times a day (52.1%), followed by 44.8% favoring brushing two times, which was the rule in daily practice (78.1%). Girls (18.9%) tended to brush teeth three times more often than boys (11.6%). There were no age differences. In contrast, in the knowledge section, boys (57.7%) and younger children (53.3%) again exhibited healthier (brushing three times) behavior than girls (50.6%) and older children (47.6%) with girls nearly equally voting for tooth brushing two (47.6%) or three times. Again, knowledge and behavior were inconsistent, with girls implementing their knowledge better in daily practice (see Table 1, Figure 1 and Overall results in Supplementary Material). The correlation analysis across all results with the Pearson correlation (knowledge correlated against behavior) showed a significant correlation (*p* < 0.05). 

## 4. Discussion

This cross-sectional questionnaire-based interview-style study in children, young adolescents and adults aged 3–19 years collected data about nutritional and hygiene knowledge and self-reported behavior. To our knowledge, this is the first study to draw a direct comparison, with such a wide age range, between knowledge and daily practice without intervention.

The voluntary nature of participation, as well as the children’s motivation and positive attitudes were advantages of our research approach. 

At the same time, this could also involve a positive selection bias and our cohort may not be a representative one. Nevertheless, it should be considered that discrepancies were also evident in this cohort, which would most likely be even more pronounced in representative groups. The topic should, therefore, be pursued in further studies with controlled demographic details. 

Children aged 7–10 years accounted for the largest proportion of our pediatric cohort. It is probable that this age group had the greatest interest in the “Long Night of Science” oral health event. This may indicate that oral hygiene education can be communicated in a playful way. Lastly, children aged 2–5 years had difficulty fully answering the questions despite parental assistance, and therefore may have been influenced by their parents.

The truthfulness of the statements (self-reported behavior) could not be verified. Since there were clear differences between behavior and knowledge, the truthfulness of statements can be assumed.

Since the questionnaire was validated in 2016 and partially modified after review for 2017 and 2018, not all questions were asked in all years, resulting in a smaller number of respondents in some cases. Certainly, with the number of participants being higher, answers would have been slightly different in these cases and, therefore, must be regarded with a certain restraint.

In 2019, Vozza et al. recorded clinical data and nutritional behavior, learning that more than 80% of the 8-year-old children studied consumed sweets or sweet drinks every day [12]. Tsang et al. investigated the nutrition and oral health status of 6-month-old to 6-year-old children in Nepal and found similar results. Though most mothers were informed about sweets causing caries, half of the children were given those daily [13]. In Croatia, Milosavljević et al. investigated the nutritional knowledge and dietary behavior of a high-school population (mean age 17.9 years). It was found that 42.3% self-reported consuming sweets three times or more per day [14]. Probably, the lower percentage was determined due to a higher mean age of the participants included. In any case, there was a clear difference in the sweets consumption of children, confirming our results. In our study, nearly all children in the first and mixed dentition phase (3–10 years) and older children (over 11 years) self-reported eating sweets on a daily basis with younger children showing better knowledge than older children. Generally, the majority of participants self-reported the daily consumption of sweets with significantly less stating that this was correct.

Jiménez-Cruz et al. found out fruits were often consumed too rarely with high-fat snacks being preferred among Mexican fifth- and ninth-grade school students [15]. Beets et al. investigated an in- and out-of-school-time program where children (5–10 years) were offered sugar-sweetened snacks and fruits with sugar-sweetened snacks being even more clearly preferred than in our study (fruits, 6%; sugar-sweetened snacks, 58%) [16]. Possibly, this is due to a positive participant selection. In our study, in all age and gender groups, fruits were considered most frequently as a suitable healthy snack with significantly fewer participants eating them. Very few participants stated that sweets were healthy with significantly more consuming them as a snack. Boys and older children were below average for the consumption of fruits and over average for the consumption of sweets. 

Vatanparast et al. checked the snack consumption behavior of Canadians finding out that 64% of 2–5-year-old children and 61% of 6–12-year-old children were eating snacks 2–3 times with 19% each eating even four times/day or more. It was found that 54% of 13–18-year-old young adolescents were consuming a snack 2–3 times/day with 11% consuming a snack four times/day or more [17]. The results are in line with our findings. The risk of caries increases significantly with more than four sugary snacks/day [18,19,20]. Generally, in our study, two trends emerged with no major differences in age and gender: one group consuming snacks always/usually (more than four times/day) and another sometimes/seldom (less than four times/day). Girls tended to consume a snack more frequently than boys, who were more cautious and better informed. 

In our study, SBBs were by far the most consumed. In contrast, the vast majority stated that water and tea should be preferred, pointing out a clear discrepancy between knowledge and behavior. Boys self-reported drinking more SBBs than girls, who in turn self-reported drinking more water and tea. Older children seemed to prefer healthier drinks than younger children, posing a potential risk to the caries-prone first dentition. The majority stated drinking sweet drinks less than every day with the majority also affirming this behavior. Nevertheless, 34.2% of participants still self-reported drinking sweet drinks every day. A clear discrepancy between boys and girls was found in their behavior, though no great differences were detected in the knowledge section. The knowledge and behavior coincided best in older children and girls. Gui et al. investigated sugar-sweetened beverage consumption and the risks of obesity and hypertension in Chinese children and adolescents in a national cross-sectional analysis finding out that 66.6% of participants consumed sugar-sweetened beverages and that 9.6% consumed these beverages more than seven times/week. In both groups, boys were consuming more sugar-sweetened beverages than girls [21]. The results confirm our findings. In our study, boys consumed more SBBs than girls. However, in our case, significantly more children stated drinking SBBs every day. We could not find studies comparing nutritional knowledge and behavior. Nevertheless, many studies discuss the link between increasing obesity, caries, and soft/sweet drinks [22,23,24]. 

While a large majority favored brushing teeth after eating sweets and a minority doing nothing, no special oral hygiene measures in daily practice were conducted in the majority. Younger children and boys seemed to have better knowledge about after-sweet-consumption behavior than older children and girls, but more often self-reported doing nothing. Surprisingly, boys more often favored chewing gums and declined doing nothing in the knowledge section. Generally, knowledge and daily practice behavior were not congruent in our study. To the authors knowledge, there are no other studies that have previously investigated immediate behavior after sweet consumption. 

Vallejos-Sánchez et al. showed that girls and older children are brushing more frequently, confirming our results [25]. Nevertheless, we could not detect differences between younger and older children (children aged 6–12). Honkala et al. investigated trends in toothbrushing in 20 countries/regions from 1994 to 2010 also finding out girls had a higher prevalence of brushing teeth more than once/day than boys in all countries/regions. The prevalence of more-than-once-a-day brushing frequency increased from 30–62% (1994) to 50–72% (2010) [26]. The study confirms our results, although our toothbrushing frequency was even higher, possibly due to a positive participant selection. Generally, in our study, a slim majority favored brushing teeth three times a day, followed by 44.8% favoring two times, which was the rule in daily practice. Boys and younger children again found the healthier (brushing three times) behavior better than girls and older children, with girls nearly equally voting for a two or three-time brushing frequency. Again, knowledge and behavior did not coincide, with girls implementing their knowledge better in daily practice.

Mirmiran et al. surveyed 7669 adolescents with a mean age of 14 ± 1 year about their nutritional knowledge and their daily practice, confirming our results, with girls having better knowledge and boys showing better behavior, contrary to our results [27]. Other studies described poorer nutritional knowledge of children (mean age: 11.2) and young adolescents (mean age: 12.5–17.5) [28,29]. Presumably, the better results in our study are due to a positive selection bias of the participants. In summary, the nutritional knowledge in all age groups was profound, but not congruent with behavior.

Poor nutrition not only affects oral health, but also general health by leading to obesity. Although we believe obesity and poor nutritional behavior to be a phenomenon of modernity, Hippocrates stated sudden death to be more frequent in fat people than lean ones [30]. Like dental caries, obesity influences quality of life and alters physical and psychosocial health. Furthermore, there is an increasing risk of becoming obese in adulthood when suffering from multiple diseases, e.g., diabetes and cardiovascular problems [9]. Various factors influence the development of obesity (e.g., behavior, genes, the environment), while weight reduction counteracts it and alleviates its consequences. There are different ways to influence weight loss: sports, medications, lifestyle modification programs, surgery and diets [31]. The benefit of several lifestyle interventions to reduce overweight and obesity in children has been closely studied [10]. 

Younger children often showed better oral hygiene knowledge than older children in our study. Possibly, this is due to the help of parents when filling out the questionnaire. Furthermore, nutritional knowledge and behavior are more controlled in younger children. Therefore, they have better feedback and thus knowledge. Nevertheless, older children often showed better behavior despite having poorer knowledge. Due to their higher age, they may be able to put their knowledge better and more consistently into practice. Younger children often showed worse behavior (e.g.: the self-reported consumption of sweets more often than older children). Certainly, parents assume their children are consuming few sweets, but children cleverly evade control. In summary, the results of this study suggest that our null hypothesis (no differences between knowledge and behavior) must be rejected.

## 5. Conclusions

CAYAs’ oral hygiene/nutritional knowledge and behavior was profound, but the translation into practice showed deficiencies. Building up controlled motivation training to encourage CAYAs (especially boys, younger children and their parents) to put their knowledge into practice is necessary. 

## Figures and Tables

**Table 1:** Summary of responses. Correlation analysis across all results with Pearson correlation (knowledge correlated against behavior) was made and a significant (*) correlation was found (*p* < 0.05).

Variables	All Years	
	Nutritional Knowledge	Nutritional Behavior
All children	328 (100%)	319 (100%)
Frequency of eating sweets *		
Every day/less than once a day	226 (68.9%)	315 (98.7%)
Never	100 (30.5%)	4 (1.3%)
Other	1 (0.003%)	0
Missing	1 (0.003%)	0
Snacks *		
Fruit	225 (68.6%)	37 (39.8%)
Sweets	27 (8.2%)	24 (25.8%)
Nothing	24 (7.3%)	9 (9.7%)
Fruit yoghurt/bread/pretzels	30 (9.2%)	12 (13.0%)
Dental care chewing gum	15 (4.6%)	3 (3.2%)
Other	7 (2.1%)	8 (8.6%)
Frequency of snacks *		
Always/usually/often	113 (48.1%)	143 (44.9%)
Sometimes/seldom	116 (49.4%)	167 (52.4%)
Never	5 (2.1%)	8 (2.5%)
Other	Not collected	1 (0.003%)
Missing	1 (0.004%)	0
Favorite/healthy drinks *		
Drinks containing sugar	59 (25.1%)	214 (67.1%)
Water/tea	175 (74.5%)	95 (29.8%)
Cocoa	1 (0.004%)	9 (2.8%)
Other	Not collected	1 (0.003%)
Frequency of sweet drinks consumption *		
Every day	57 (24.3%)	109 (34.2%)
Less than every day	175 (74.5%)	209 (65.5%)
Other	Not collected	1 (0.003%)
Missing	3 (1.3%)	0
Behavior after consumption of sweets *		
Doing nothing	18 (5.5%)	187 (58.6%)
Brushing teeth	231 (70.4%)	52 (16.3%)
Rinsing mouth with water/mouthwash	43 (13.1%)	54 (36.6%)
Chewing dental care gum	31 (9.5%)	18 (5.6%)
Chewing sweet gum	3 (0.009%)	2 (0.006%)
Other	2 (0.006%)	6 (1.9%)
Frequency of brushing teeth *		
Three times a day	171 (52.1%)	51 (16.0%)
Twice a day	147 (44.8%)	249 (78.1%)
Every day only in the morning/evening	3 (0.009%)	17 (5.3%)
Once a week	1 (0.003%)	1 (0.003%)
Other	6 (1.8%)	1 (0.003%)

## Data Availability

Data sharing not applicable. No new data were created or analyzed in this study.

## Supplementary Materials

The following supporting information can be downloaded at: https://www.mdpi.com/article/10.3390/ijerph191610055/s1, Questionnaire S1; Questionnaire S2; Table S1: Overall Results: Correlation analysis across all results with Pearson Correlation (knowledge correlated against behavior) was made and a significant (*) correlation found (*p* < 0.05).
