# Supplementary material for: Nutritional and Oral Hygiene Knowledge versus Reported Behavior of Children and Adolescents—A Cross-Sectional Interview-Based Study
Source: Int J Environ Res Public Health. 2022 Aug 15;19(16):10055. doi: 10.3390/ijerph191610055 (PMC9408341; doi:10.3390/ijerph191610055)
Supplement: Supplementary file 1 [file ijerph-19-10055-s001.zip › Questionnaire.pdf]

Questionnaire S1

Age:
□ Male
□ Female

Part One: Eating Habits

1. How often do you eat sweets?
□ daily
□ never
□ other

2. What do you eat in between the three main meals?
□ sweets
□ fruit yoghurt/bread/ pretzels
□ fruit
□ nothing
□ dental care chewing gum
□ other

3. How often do you eat something in between the three main meals?
□ always/usually (> 4 times/ day)
□ sometimes/seldom (< 4 times/ day)
□ never
□ other

4. What do you like to drink?
□ Sugar sweetened beverages (SBB)
□ cocoa
□ water/ tea (unsweetened)
□ other

5. How often do you drink sweet drinks?
□ every day
□ less than every day
□ other

6. What do you do after eating candy or drinking something sweet?
□ nothing
□ chewing sweet chewing gum
□ rinsing mouth with mouthwash
□ chewing dental care chewing gum
□ brushing teeth
□ other

7. How often do you brush your teeth?
□ once a week
□ once a day
□ twice a day
□ three times every day
□ other

Part Two: Nutritional Knowledge

8. How often can you eat sweets?
□ daily
□ never
□ other

9. What can you eat between the three main meals?
□ sweets
□ fruit yoghurt/bread/ pretzels
□ fruit
□ nothing
□ dental care chewing gum
□ other

10. What should you do after eating candy or drinking something sweet?
□ nothing
□ chewing sweet chewing gum
□ rinsing mouth with mouthwash
□ chewing dental care chewing gum
□ brushing teeth
□ other

11. How often should you brush your teeth?
□ once a week
□ once a day
□ twice a day
□ three times every day
□ other

Questionnaire S2

Age:
□ Male
□ Female

Part One: Eating Habits

1. How often do you eat sweets?
□ daily
□ never
□ other

2. How often do you eat something in between the three main meals?
□ always/usually (> 4 times/ day)
□ sometimes/seldom (< 4 times/ day)
□ never
□ other

3. What do you like to drink?
□ Sugar sweetened beverages (SBB)
□ cocoa
□ water/ tea (unsweetened)
□ other

4. How often do you drink sweet drinks?
□ every day
□ less than every day
□ other

5. What do you do after eating candy or drinking something sweet?
□ nothing
□ chewing sweet chewing gum
□ rinsing mouth with mouthwash
□ chewing dental care chewing gum
□ brushing teeth
□ other

7. How often do you brush your teeth?
□ once a week
□ once a day
□ twice a day
□ three times every day
□ other

Part Two: Nutritional Knowledge

8. How often can you eat sweets?
□ daily
□ never
□ other

9. What can you eat between the three main meals?
□ sweets
□ fruit yoghurt/bread/ pretzels
□ fruit
□ nothing
□ dental care chewing gum
□ other

10. How often can you eat something between the three main meals?
□ always/usually (> 4 times/ day)
□ sometimes/seldom (< 4 times/ day)
□ never
□ other

11. What should you drink?
□ Sugar sweetened beverages (SBB)
□ cocoa
□ water/ tea (unsweetened)
□ other

12. How often should you drink sweet drinks?
□ every day
□ less than every day
□ other

13. What should you do after eating candy or drinking something sweet?
□ nothing
□ chewing sweet chewing gum
□ rinsing mouth with mouthwash
□ chewing dental care chewing gum
□ brushing teeth
□ other

14. How often should you brush your teeth?
□ once a week
□ once a day
□ twice a day
□ three times every day
□ other
