# Supplementary material for: Nutritional and Oral Hygiene Knowledge versus Reported Behavior of Children and Adolescents—A Cross-Sectional Interview-Based Study
Source: Int J Environ Res Public Health. 2022 Aug 15;19(16):10055. doi: 10.3390/ijerph191610055 (PMC9408341; doi:10.3390/ijerph191610055)
Supplement: Supplementary file 1 [file ijerph-19-10055-s001.zip › Table Supplement.pdf]

<u>**Variables**</u>	Questionnaire Year 2016		Questionnaire Year 2017		Questionnaire Year 2018		All Years	
	Nutritional Knowledge	Nutritional Behaviour	Nutritional Knowledge	Nutritional Behaviour	Nutritional Knowledge	Nutritional Behaviour	Nutritional Knowledge	Nutritional Behaviour
<u>**All children**</u>	**93 (100%)**		**125 (100%)**	**115 (100%)**	**110 (100%)**	**111 (100%)**	**328 (100%)**	**319 (100%)**
<u>***Gender***</u>								
Boys	*38 (40.9%)*		*36 (28.8%)*	*46 (40.0%)*	*37 (33.6%)*	*37 (33.3%)*	*111 (33.8%)*	*121 (37.9%)*
Girls	*39 (41.9%)*		*65 (52.0%)*	*63 (54.8%)*	*62 (56.4%)*	*57 (51.4%)*	*166 (50.6%)*	*159 (49.8%)*
missing	*16 (17.2%)*		*24 (19.2%)*	*6 (5.2%)*	*11 (10.0%)*	*17 (15.3%)*	*51 (15.6%)*	*39 (12.2%)*
<u>**Age**</u>								
3-10yrs	53 (57.2%)		67 (53.6%)	85 (73.8%)	75 (68.1%)	66 (59.6%)	195 (59.5%)	204 (63.9%)
as of 11yrs	10 (10.8%)		17 (13.6%)	22 (19.1%)	15 (13.6%)	21 (18.9%)	42 (12.8%)	53 (16.6%)
missing	30 (32.0%)		41 (32.8%)	8 (7.0%)	20 (18.2%)	24 (21.6%)	91 (27.7%)	62 (19.4%)
<u>**Frequence of eating sweets**</u>*								
**daily**	**66 (71.1%)**	**92 (98.9%)**	**76 (60.8%)**	**114 (99.1%)**	**84 (76.4%)**	**109 (98.2%)**	**226 (68.9%)**	**315 (98.7%)**
Boys	*25 (65.8%)*	*38 (100.0%)*	*21 (58.4%)*	*46 (100.0%)*	*24 (64.8%)*	*36 (97.3%)*	*70 (63.1%)*	*120 (99.1%)*
Girls	*27 (69.2%)*	*38 (97.4%)*	*39 (60.0%)*	*62 (98.4%)*	*51 (82.3%)*	*57 (100.0%)*	*117 (70.5%)*	*157 (98.7%)*
3-10 yr olds	36 (68.0%)	53 (100.0%)	42 (62.7%)	84 (98.9%)	56 (74.7%)	65 (98.5%)	134 (68.7%)	202 (99.0%)
as of 11yrs old	9 (90.0%)	10 (100.0%)	9 (52.9%)	22 (100.0%)	14 (93.3%)	21 (100.0%)	32 (76.2%)	63 (100.0%)
**never**	**26 (28.0%)**	**1 (1.1%)**	**48 (38.4%)**	**1 (0.9%)**	**26 (23.6%)**	**2 (1.8%)**	**100 (30.5%)**	**4 (1.3%)**
Boys	*12 (31.6%)*	*0*	*15 (41.7%)*	*0*	*13 (35.1%)*	*1 (2.7%)*	*40 (36.0%)*	*1 (0.008%)*
Girls	*12 (30.8%)*	*1 (2.7%)*	*26 (40.0%)*	*1 (1.6%)*	*11 (17.7%)*	*0*	*49 (29.5%)*	*2 (1.3%)*
3-10 yr olds	16 (30.2%)	0 (0%)	25 (37.3%)	1 (1.2%)	19 (25.4%)	1 (1.5%)	60 (30.8%)	2 (0.009%)
as of 11yrs old	1 (10.0%)	0 (0%)	8 (47.1%)	0	1 (6.7%)	0	10 (23.8%)	0
**other**	**1 (1.1%)**	**0**	**Not collected**	**Not collected**	**Not collected**	**Not collected**	**1 (0.003%)**	**0**
Boys	*1 (2.6%)*	*0*					*1 (0.009%)*	*0*
Girls	*0*	*0*					*0*	*0*
3-10 yr olds	1 (1.9%)	0					1 (0.005%)	0
as of 11yrs old	0	0					0	0
**missing**	**0**	**0**	**1 (0.8%)**	**0**	**0**	**0**	**1 (0.003%)**	**0**
								N = see above in 2016
<u>**Snacks**</u>*				**Not collected**		**Not collected**		
**fruit**	**61 (65.6%)**	**37 (39.8%)**	**93 (74.4%)**		**71 (64.5%)**		**225 (68.6%)**	**37 (39.8%)**
Boys	*24 (63.2%)*	*9 (23.7%)*	*24 (66.7%)*		*23 (62.2%)*		*71 (64.0%)*	*9 (23.7%)*
Girls	*26 (66.7%)*	*22 (56.4%)*	*51 (78.5%)*		*39 (62.9%)*		*116 (70.0%)*	*22 (56.4%)*
3-10 yr olds	35 (66.0%)	21 (39.6%)	50 (74.6%)		48 (64.0%)		133 (68.2%)	21 (39.6%)
as of 11yrs old	4 (40.0%)	2 (20.0%)	12 (70.6%)		9 (60.0%)		25 (59.5%)	2 (20.0%)
**sweets**	**4 (4.3%)**	**24 (25.8%)**	**11 (8.8%)**		**12 (10.9%)**		**27 (8.2%)**	**24 (25.8%)**
Boys	*3 (7.9%)*	*12 (31.8%)*	*4 (11.2%)*		*4 (10.8%)*		*11 (10.0%)*	*12 (31.8%)*
Girls	*1 (2.6%)*	*8 (20.5%)*	*4 (6.2%)*		*7 (11.3%)*		*12 (7.2%)*	*8 (20.5%)*
3-10 yr olds	4 (7.5%)	13 (24.5%)	5 (7.5%)		7 (9.4%)		16 (8.2%)	13 (24.5%)
as of 11yrs old	0	3 (30.0%)	2 (11.8%)		3 (20.0%)		5 (1.2%)	3 (30.0%)
**nothing**	**8 (8.6%)**	**9 (9.7%)**	**8 (6.4%)**		**8 (7.3%)**		**24 (7.3%)**	**9 (9.7%)**
Boys	*6 15.8%)*	*5 (13.2%)*	*4 (11.2%)*		*5 (13.5%)*		*15 (13.5%)*	*5 (13.2%)*
Girls	*1 (2.6%)*	*3 (7.7%)*	*3 (4.6%)*		*3 (4.8%)*		*7 (4.2%)*	*3 (7.7%)*
3-10 yr olds	4 (7.5%)	7 (13.2%)	5 (7.5%)		6 (8.0%)		15 (7.7%)	7 (13.2%)
as of 11yrs old	2 (20.0%)	0	1 (5.9%)		0		3 (7.1%)	0
**fruit yoghurt/bread/pretzels**	**11 (11.8%)**	**12 (13.0%)**	**8 (6.4%)**		**11 (10.0%)**		**30 (9.2%)**	**12 (13.0%)**
Boys	*4 (10.5%)*	*6 (15.8%)*	*3 (8.4%)*		*2 (5.4%)*		*9 (8.1%)*	*6 (15.8%)*
Girls	*4 (10.3%)*	*3 (7.7%)*	*4 (6.1%)*		*8 (13.2%)*		*16 (9.6%)*	*3 (7.7%)*
3-10 yr olds	5 (9.4%)	8 (15.1%)	4 (6.0%)		8 (10.7%)		17 (8.7%)	8 (15.1%)
as of 11yrs old	2 (20.0%)	1 (10.0%)	1 (5.9%)		2 (13.3%)		5 (11.9%)	1 (10.0%)
**dental care chewing gum**	**2 (2.2%)**	**3 (3.2%)**	**5 (4.0%)**		**8 (7.3%)**		**15 (4.6%)**	**3 (3.2%)**
Boys	*0*	*1 (2.6%)*	*1 (2.8%)*		*3 (8.1%)*		*4 (3.6%)*	*1 (2.6%)*
Girls	*2 (5.1%)*	*0*	*3 (4.6%)*		*5 (8.1%)*		*10 (6.0%)*	*0*
3-10 yr olds	0	1 (1.9%)	3 (4.5%)		6 (8.0%)		9 (4.6%)	1 (1.9%)
as of 11yrs old	1 (10.0%)	0	1 (5.9%)		1 (6.7%)		3 (7.1%)	0
**other**	**7 (7.5%)**	**8 (8.6%)**	**Not collected**		**Not collected**		**7 (2.1%)**	**8 (8.6%)**
Boys	*1 (2.6%)*	*5 (13.2%)*					*1 (0.009%)*	*5 (13.2%)*
Girls	*5 (12.8%)*	*3 (7.9%)*					*5 (3.0%)*	*3 (7.9%)*
3-10 yr olds	5 (9.4%)	3 (5.7%)					5 (2.7%)	3 (5.7%)
as of 11yrs old	1 (10.0%)	3 (30.0%)					1 (2.4%)	3 (30.0%)
							N= 2017+2018 (no values from 2016 included)	
<u>**Frequence of snacks**</u>*	**Not collected**							
**always/usually (>4 times/day)**		**42 (45.2%)**	**63 (50.4%)**	**59 (51.3%)**	**50 (45.5%)**	**42 (37.8%)**	**113 (48.1%)**	**143 (44.9%)**
Boys		*14 (36.8%)*	*15 (41.7%)*	*26 (65.5%)*	*17 (46.0%)*	*10 (27.0%)*	*32 (43.8%)*	*50 (41.3%)*
Girls		*22 (56.5%)*	*36 (55.4%)*	*29 (46.0%)*	*31 (50.0%)*	*25 (43.9%)*	*67 (52.8%)*	*76 (47.8%)*
3-10 yr olds		22 (41.5%)	35 (52.2%)	42 (49.4%)	36 (48.0%)	25 (37.9%)	71 (50.0%)	89 (43.6%)
as of 11yrs old		5 (50.9%)	8 (47.1%)	12 (54.5%)	7 (46.7%)	8 (44.5%)	15 (46.9%)	25 (47.1%)
**sometimes/ seldom (<4 times/day)**		**48 (51.7%)**	**61 (48.8%)**	**52 (45.2%)**	**55 (50.0%)**	**67 (60.4%)**	**116 (49.4%)**	**167 (52.4%)**
Boys		*23 (60.5%)*	*21 (58.4%)*	*18 (39.1%)*	*19 (51.4%)*	*25 (67.6%)*	*40 (54.8%)*	*66 (54.5%)*
Girls		*15 (38.5%)*	*28 (43.1%)*	*32 (50.8%)*	*30 (48.4%)*	*32 (56.1%)*	*58 (45.7%)*	*79 (49.7%)*
3-10 yr olds		28 (52.8%)	32 (47.8%)	39 (45.9%)	37 (49.4%)	41 (62.1%)	69 (48.6%)	108 (52.9%)
as of 11yrs old		5 (50.0%)	9 (52.9%)	10 (45.5%)	8 (53.4%)	11 (61.1%)	17 (53.1%)	26 (49.1%)
**never**		**2 (2.2%)**	**1 (0.8%)**	**4 (3.5%)**	**4 (3.6%)**	**2 (1.8%)**	**5 (2.1%)**	**8 (2.5%)**
Boys		*1 (2.6%)*	*0*	*2 (4.4%)*	*1 (2.7%)*	*2 (5.4%)*	*1 (1.4%)*	*5 (4.1%)*
Girls		*1 (2.6%)*	*1 (1.5%)*	*2 (3.2%)*	*1 (1.6%)*	*0*	*2 (1.6%)*	*3 (1.9%)*
3-10 yr olds		2 (3.8%)	0	4 (4.7%)	2 (2.7%)	0	2 (1.4%)	6 (2.9%)
as of 11yrs old		0	0	0	0	2 (11.1%)	0	2 (3.8%)

**other**		**1 (1.1%)**	**Not collected**	**Not collected**	**Not collected**	**Not collected**	**Not collected**	**1 (0.003%)**
Boys		*0*	*0*	*0*				*0*
Girls		*1 (2.6%)*	*0*	*0*				*1 (0.006%)*
3-10 yr olds		1 (1.9%)		0	0			1 (0.005%)
as of 11yrs old		0	0		0	0		0
**missing**		**0**	**0**	**0**	**1 (0.9%)**	**0**	**1 (0.004%)**	**0**
**Favorite/healthy drinks***								
**Sugar sweetened beverage (SBB)**	**Not collected**	**63 (67.7%)**	**37 (29.6%)**	**88 (76.5%)**	**22 (20.0%)**	**63 (56.8%)**	**59 (25.1%)**	**214 (67.1%)**
Boys		*26 (68.4%)*	*14 (38.9%)*	*36 (78.3%)*	*4 (10.8%)*	*24 (64.9%)*	*18 (24.7%)*	*86 (71.1%)*
Girls		*25 (64.1%)*	*15 (23.1%)*	*48 (76.2%)*	*14 (22.6%)*	*31 (54.4%)*	*29 (22.9%)*	*104 (65.4%)*
3-10 yr olds		36 (67.9%)	17 (25.4%)	69 (81.2%)	17 (22.7%)	40 (60.6%)	34 (23.9%)	145 (71.1%)
as of 11yrs old		6 (60.0%)	3 (17.7%)	13 (59.1%)	1 (6.7%)	13 (61.9%)	4 (12.5%)	32 (60.4%)
**water/tea (unsweetened)**		**24 (25.8%)**	**87 (69.6%)**	**25 (21.7%)**	**88 (80.0%)**	**46 (41.4%)**	**175 (74.5%)**	**95 (29.8%)**
Boys		*9 23.7%)*	*22 (61.2%)*	*10 (21.7%)*	*33 (89.2%)*	*12 (32.4%)*	*55 (75.3%)*	*31 (25.6%)*
Girls		*12 (30.8%)*	*50 (76.9%)*	*13 (20.6%)*	*48 (77.4%)*	*26 (45.6%)*	*98 (77.2%)*	*51 (32.1%)*
3-10 yr olds		12 (22.6%)	50 (74.6%)	15 (17.6%)	58 (77.4%)	25 (37.9%)	108 (76.1%)	52 (25.5%)
as of 11yrs old		4 (40.0%)	14 (82.4%)	8 (36.4%)	14 (93.4%)	8 (38.1%)	28 (87.5%)	20 (37.7%)
**cocoa**		**5 (5.4%)**	**1 (0.8%)**	**2 (1.7%)**	**0**	**2 (1.8%)**	**1 (0.004%)**	**9 (2.8%)**
Boys		*3 (7.9%)*	*0*	*0*	*0*	*1 (2.7%)*	*0*	*4 (3.3%)*
Girls		*1 (2.6%)*	*0*	*2 (3.2%)*	*0*	*0*	*0*	*3 (1.9%)*
3-10 yr olds		4 (7.5%)	0	1 (1.2%)	0	1 (1.5%)	0	6 (2.9%)
as of 11yrs old		0	0	1 (4.5%)	0	0	0	1 (1.9%)
**other**		**1 (1.1%)**	**Not collected**	**Not collected**	**Not collected**	**Not collected**	**Not collected**	**1 (0.003%)**
Boys		*0*						*0*
Girls		*1 (2.6%)*						*1 (0.006%)*
3-10 yr olds		1 (1.9%)						1 (0.005%)
as of 11yrs old		0						0
**Frequence of sweet drinks**	**Not collected**							
**consumption***		**37 (39.8%)**	**28 (22.4%)**	**39 (33.9%)**	**29 (26.3%)**	**33 (29.7%)**	**57 (24.3%)**	**109 (34.2%)**
**every day**		*17 (44.7%)*	*10 (27.8%)*	*20 (43.5%)*	*8 (21.6%)*	*15 (40.5%)*	*18 (24.7%)*	*52 (43.0%)*
Boys		*12 (30.8%)*	*14 (21.5%)*	*17 (27.0%)*	*17 (27.4%)*	*14 (24.6%)*	*31 (24.4%)*	*43 (27.0%)*
Girls		19 (35.8%)	14 (20.9%)	31 (36.5%)	16 (21.3%)	23 (34.8%)	30 (21.1%)	73 (35.8%)
3-10 yr olds		5 (50.0%)	4 (23.5%)	5 (22.7%)	7 (46.7%)	6 (28.6%)	11 (34.4%)	16 (30.2%)
as of 11yrs old		**55 (59.2%)**	**94 (75.2%)**	**76 (66.0%)**	**81 (73.6%)**	**78 (70.3%)**	**175 (74.5%)**	**209 (65.5%)**
**less than every day**		*21 (55.3%)*	*26 (72.3%)*	*26 (56.6%)*	*29 (78.4%)*	*22 (59.5%)*	*55 (75.4%)*	*69 (57.0%)*
Boys		*26 (66.7%)*	*49 (75.4%)*	*46 (72.9%)*	*45 (72.6%)*	*43 (75.4%)*	*94 (74.0%)*	*115 (72.3%)*
Girls		33 (62.2%)	52 (77.6%)	54 (63.6%)	59 (78.6%)	43 (65.2%)	111 (78.1%)	130 (63.7%)
3-10 yr olds		5 (50.0%)	13 (76.5%)	17 (77.3%)	8 (53.4%)	15 (71.5%)	21 (65.7%)	37 (69.8%)
as of 11yrs old		**1 (1.1%)**	**Not collected**	**Not collected**	**Not collected**	**Not collected**	**Not collected**	**1 (0.003%)**
**other**		*0*						*0*
Boys		*1 (2.6%)*						*1 (0.006%)*
Girls		1 (1.9%)						1 (0.005%)
3-10 yr olds		0						0
as of 11yrs old		**0**	**3 (2.4%)**	**0**	**0**	**0**	**3 (1.3%)**	**0**
**missing**			*2 (3.1%)*				*2 (1.6%)*	
Girls			1 (1.5%)				1 (0.007%)	
3-10 yr olds								
**Behavior after consumption of sweets***								
**doing nothing**	**7 (7.5%)**	**63 (67.7%)**	**5 (4.0%)**	**63 (54.8%)**	**6 (5.5%)**	**61 (55.0%)**	**18 (5.5%)**	**187 (58.6%)**
Boys	*1 (2.6%)*	*29 (76.3%)*	*1 (2.8%)*	*28 (60.9%)*	*2 (5.4%)*	*22 (59.5%)*	*4 (3.6%)*	*79 (65.3%)*
Girls	*3 (7.7%)*	*23 (59.0%)*	*3 (4.6%)*	*32 (50.8%)*	*4 (6.5%)*	*35 (61.4%)*	*10 (6.0%)*	*90 (56.6%)*
3-10 yr olds	4 (7.6%)	36 (67.9%)	3 (4.5%)	47 (55.3%)	4 (5.4%)	42 (63.6%)	11 (5.6%)	125 (61.3%)
as of 11yrs old	0	5 (50.0%)	1 (5.9%)	12 (54.5%)	2 (13.4%)	12 (57.1%)	3 (7.1%)	29 (54.7%)
**brushing teeth**	**68 (73.1%)**	**12 (12.9%)**	**83 (66.4%)**	**16 (13.9%)**	**80 (72.7%)**	**24 (21.6%)**	**231 (70.4%)**	**52 (16.3%)**
Boys	*29 (76.3%)*	*2 (6.3%)*	*26 (72.3%)*	*6 (13.0%)*	*27 (73.0%)*	*9 (24.3%)*	*82 (73.9%)*	*17 (14.1%)*
Girls	*29 (74.4%)*	*7 (18.0%)*	*41 (63.1%)*	*10 (15.9%)*	*44 (71.0%)*	*11 (19.3%)*	*114 (68.7%)*	*28 (17.6%)*
3-10 yr olds	41 (77.4%)	5 (9.4%)	48 (71.6%)	12 (14.1%)	55 (73.4%)	15 (22.7%)	144 (73.9%)	32 (15.7%)
as of 11yrs old	8 (80.0%)	2 (20.0%)	8 (47.1%)	3 (13.6%)	9 (60.0%)	4 (19.1%)	25 (59.5%)	9 (17.0%)
**rinsing mouth with mouthwash**	**6 (6.4%)**	**7 (7.5%)**	**23 (18.4%)**	**29 (25.2%)**	**14 (12.8%)**	**18 (16.2%)**	**43 (13.1%)**	**54 (36.6%)**
Boys	*2 (5.3%)*	*4 (10.5%)*	*3 (8.4%)*	*8 (17.4%)*	*3 (8.1%)*	*4 (10.8%)*	*8 (7.2%)*	*16 (13.2%)*
Girls	*2 (5.1%)*	*3 (7.7%)*	*17 (26.6%)*	*18 (28.6%)*	*9 (14.6%)*	*9 (15.8%)*	*28 (16.8%)*	*30 (18.8%)*
3-10 yr olds	3 (5.7%)	5 (9.4%)	9 (13.4%)	21 (24.7%)	8 (10.7%)	6 (9.1%)	20 (10.3%)	32 (15.7%)
as of 11yrs old	0	*1 (10.0%)*	7 (41.2%)	5 (22.7%)	2 (13.4%)	5 (23.9%)	9 (21.4%)	11 (20.8%)
**chewing dental care gum**	**9 (9.7%)**	**5 (5.4%)**	**12 (9.6%)**	**6 (5.2%)**	**10 (9.1%)**	**7 (6.3%)**	**31 (9.5%)**	**18 (5.6%)**
Boys	*5 (13.2%)*	*1 (2.6%)*	*5 (13.9%)*	*3 (6.5%)*	*5 (13.5%)*	*1 (2.7%)*	*15 (13.5%)*	*5 (4.1%)*
Girls	*3 (7.7%)*	*2 (5.1%)*	*3 (4.6%)*	*3 (4.8%)*	*5 (8.1%)*	*2 (3.5%)*	*11 (6.6%)*	*7 (4.4%)*
3-10 yr olds	3 (5.7%)	2 (3.8%)	6 (9.0%)	4 (4.7%)	8 (10.7%)	2 (3.0%)	17 (8.7%)	8 (3.9%)
as of 11yrs old	1 (10.0%)	1 (10.0%)	1 (5.9%)	2 (9.1%)	2 (13.4%)	0	4 (9.5%)	3 (5.7%)
**chewing sweet gum**	**1 (1.1%)**	**0**	**2 (1.6%)**	**1 (0.9%)**	**0**	**1 (0.9%)**	**3 (0.009%)**	**2 (0.006%)**
Boys	*0*	*0*	*1 (2.8%)*	*1 (2.2%)*	*0*	*1 (2.7%)*	*1 (0.009%)*	*2 (1.7%)*
Girls	*1 (2.6%)*	*0*	*1 (1.5%)*	*0*	*0*	*0*	*2 (1.2%)*	*0*
3-10 yr olds	0	0	1 (1.5%)	1 (1.2%)	0	1 (1.5%)	1 (0.005%)	2 (0.01%)
as of 11yrs old	1 (10.0%)	0	0	0	0	0	1 (2.4%)	0
**other**	**2 (2.2%)**	**6 (6.5%)**	**Not collected**	**Not collected**	**Not collected**	**Not collected**	**2 (0.006%)**	**6 (1.9%)**
Boys	*1 (2.6%)*	*2 (5.3%)*					*1 (0.009%)*	*2 (1.7%)*
Girls	*1(2.6%)*	*4 (10.3%)*					*1 (0.006%)*	*4 (2.5%)*
3-10 yr olds	2 (3.8%)	5 (9.4%)					2 (1.0%)	5 (2.5%)
as of 11yrs old	0	1 (10.0%)					0	1 (1.9%)
**Frequence of brushing teeth***								
**three times a day**	**50 (53.8%)**	**17 (18.3%)**	**74 (59.2%)**	**20 (17.4%)**	**47 (42.7%)**	**14 (12.6%)**	**171 (52.1%)**	**51 (16.0%)**
Boys	*22 (57.9%)*	*7 (18.4%)*	*22 (61.2%)*	*4 (8.7%)*	*20 (54.1%)*	*3 (8.1%)*	*64 (57.7%)*	*14 (11.6%)*
Girls	*21 (53.9%)*	*8 (20.5%)*	*42 (64.6%)*	*16 (25.4%)*	*21 (33.9%)*	*6 (10.5%)*	*84 (50.6%)*	*30 (18.9%)*
3-10 yr olds	24 (45.3%)	9 (17.0%)	45 (67.2%)	17 (20.0%)	35 (46.7%)	6 (9.1%)	104 (53.3%)	32 (15.7%)
as of 11yrs old	8 (80.0%)	2 (20.0%)	10 (58.8%)	3 (13.6%)	2 (13.4%)	3 (14.3%)	20 (47.6%)	8 (15.1%)

**twice a day**	**34 (36.6%)**	**68 (73.1%)**	**51 (40.8%)**	**90 (78.3%)**	**62 (56.4%)**	**91 (82.0%)**	**147 (44.8%)**	**249 (78.1%)**
Boys	*13 (34.2%)*	*29 (76.3%)*	*14 (38.9%)*	*41 (89.1%)*	*16 (43.2%)*	*30 (81.1%)*	*43 (38.7%)*	*100 (82.6%)*
Girls	*15 (38.5%)*	*25 (64.1%)*	*23 (35.4%)*	*43 (68.3%)*	*41 (66.1%)*	*50 (87.7%)*	*79 (47.6%)*	*118 (74.2%)*
3-10 yr olds	23 (43.4%)	39 (73.6%)	22 (32.8%)	65 (76.5%)	39 (52.0%)	57 (86.4%)	84 (43.1%)	161 (78.9%)
as of 11yrs old	2 (20.0%)	6 (60.0%)	7 (41.2%)	18 (82.0%)	13 (86.7%)	17 (81.0%)	22 (52.4%)	41 (77.4%)
**once a day**	**3 (3.3%)**	**6 (6.5%)**	**0**	**5 (4.3%)**	**0**	**6 (5.4%)**	**3 (0,009%)**	**17 (5.3%)**
Boys	*2 (5.2%)*	*1 (2.6%)*	*0*	*1 (2.2%)*	*0*	*4 (10.8%)*	*2 (0.02%)*	*6 (5.0%)*
Girls	*0*	*5 (12.8%)*	*0*	*4 (6.3%)*	*0*	*1 (1.8%)*	*0*	*10 (6.3%)*
3-10 yr olds	2 (3.8%)	4 (7.6%)	0	3 (5.7%)	0	3 (4.5%)	2 (0.01%)	10 (4.9%)
as of 11yrs old	0	1 (10.0%)	0	1 (4.5%)	0	1 (4.8%)	0	3 (5.7%)
**once a week**	**0**	**1 (1.1%)**	**0**	**0**	**1 (0.9%)**	**0**	**1 (0.003%)**	**1 (0.003%)**
Boys	*0*	*1 (2.6%)*	*0*	*0*	*1 (2.7%)*	*0*	*1 (0.009%)*	*1 (0.008%)*
Girls	*0*	*0*	*0*	*0*	*0*	*0*	*0*	*0*
3-10 yr olds	0	0	0	0	1 (1.4%)	0	1 (0.005%)	0
as of 11yrs old	0	1 (10.0%)	0	0	0	0	0	1 (1.9%)
**other**	**6 (6.5%)**	**1 (1.1%)**	**Not collected**	**Not collected**	**Not collected**	**Not collected**	**6 (1.8%)**	**1 (0.003%)**
Boys	*1 (2.6%)*	*0*					*1 (0.009%)*	*0*
Girls	*3 (7.7%)*	*1 (2.6%)*					*3 (1.8%)*	*1 (0.006%)*
3-10 yr olds	4 (7.6%)	1 (1.9%)					4 (2.1%)	1 (0.005%)
as of 11yrs old	0	0					0	0

Table S1: Overall results: Correlation analysis across all results with Pearson Correlation (knowledge correlated against behavior) was made and a significant (*) correlation found (p<0.05).
